# The Physiological Effects of the Franklinic Current

**Published:** 1893-08

**Authors:** 


THE PHYSIOLOGICAL EFFECTS OF THE FRANKLINIC CURRENT.—Damian of Paris has recently published a study upon the above subject. By means of his experiments he has conclusively demonstrated that under positive electrization the heart's action is stimulated and contracts with greater energy, as indicated by sphygmographic tracings. The temperature, with the same insulation, and with sparks, was increased from 0.025 to 0.4 of a degree; while with the simple bath, positive insulation, there was an increase in temperature of 0.5 of a degree.

The production of urea under the influence of the electropositive bath was sensibly increased, its weight being elevated from 24.64 to 26.3 grammes. The same insulation with sparks increased the daily total of urea from 27 to 28, 30, 31, and 32.68 grammes. The electro-negative bath was followed by an increase in the volume of urine. In no other form of application was the volume sensibly increased or diminished, while under the same insulation with sparks the amount of urea was progressively diminished; but less with bath. The phosphoric acid in the urine was decreased under the negative electrization, but more so with the positive charge.

The alkaline phosphates were diminished in the same proportion, but more so with positive than with negative; less with bath than with spark. The earthy phosphates were decreased under negative, less under positive electrization.

Thus we see by M. Damian's experiments that the heart's action is stimulated, the circulation improved, the temperature augmented, and the excretions increased. Further than this he has not yet demonstrated; but he believes that there are effects which are as yet undemonstrable. A moral or psychic effect is universally conceded; but the above experiments have clearly proven that the influence of static electricity does not end there, as many are disposed to claim.

